# Esthetic evaluation of implant-supported single crowns: a comparison of objective and patient-reported outcomes

**DOI:** 10.1186/s40729-018-0153-3

**Published:** 2019-01-07

**Authors:** Mehmet Ali Altay, Alper Sindel, Hüseyin Alican Tezerişener, Nelli Yıldırımyan, Mehmet Mustafa Özarslan

**Affiliations:** 1 Department of Oral and Maxillofacial Surgery, Faculty of Dentistry, Akdeniz University, Dumlupinar Boulevard, Campus, 07058 Antalya, Turkey; 2 Department of Prosthodontics, Faculty of Dentistry, Akdeniz University, Antalya, Turkey

**Keywords:** Dental implants, Esthetics, Soft tissue, Single implant, Anterior maxilla

## Abstract

**Background:**

This study investigated objective and patient-reported esthetic outcomes and their correlation for single-tooth implant restorations in the maxillary anterior region.

**Methods:**

Nineteen patients were included. Gingival biotypes and smile lines were evaluated. Esthetic evaluation was performed according to the pink and white esthetic scores (PES and WES). Patients rated their satisfaction regarding the implant treatment using a subjective outcome questionnaire and a 10-cm visual analogue scale (VAS).

**Results:**

The mean PES and WES were 10.7 (range 5–13, SD ± 2.24) and 8.6 (range 8–10, SD ± 0.60), respectively. The overall mean VAS was 8.54 ± 0.36 (range 3.8–9.8). No significant correlation was found between VAS and PES or WES (*p* = 0.475, *p* = 0.984, respectively). PES and WES scores for gingival biotypes failed to show any statistically significant difference (*p* = 0.701, *p* = 0.831). There was a significant negative correlation between the smile line and VAS; indicating that patients with lower smile lines expressed higher patient satisfaction (*p* = .001).

**Conclusions:**

Professionally reported esthetic outcomes (PES and WES results) may not correlate with patient-reported outcomes. Smile line is a significant factor in patient satisfaction, which should be evaluated thoroughly prior to implant placement in the anterior maxilla.

## Background

Rehabilitation of missing teeth in the anterior maxilla with an implant-supported fixed prosthesis is a widely accepted treatment modality [1]. Dental implants have high rates of predictability in terms of osseointegration, particularly due to improvements in treatment techniques and surface topography [2]. However, rehabilitation with dental implants is not yet considered a perfect treatment modality as several problems may be encountered during and after implant placement [3]. The success of an implant-supported prosthesis is dependent on several factors and classically defined according to criteria, which mainly focus on osseointegration and the amount of radiographic bone loss [4, 5]. Although these factors are indispensable elements of implant success, they often fail to objectively evaluate all aspects of treatment outcomes, particularly of the implants placed in esthetically demanding areas. In our day, dental implants are commonly used not just to provide patients with function but also form and esthetics. Therefore, an accurate assessment of success inevitably involves objective and patient-reported esthetic evaluation of the treatment outcomes.

An early attempt to evaluate the esthetic aspects of dental implants was made in 1997 by Torsten Jemt who proposed a papilla index, which assessed the size of the interproximal papilla [6]. Since then, successively described evaluation methods of esthetic outcomes have been subject to several studies aiming to test the accuracy and effectiveness of these methods. Today, several factors in addition to the size of the interproximal papilla including the color, form, and the level of peri-implant soft tissues have been included in evaluation of the esthetic outcomes [7, 8].

Recently, more objective approaches to evaluating esthetics of single implant restorations have been described. The pink esthetic score (PES), which evaluates soft tissue esthetics around an implant, was introduced by Furhauser et al. [9]. This effort was followed by Belser et al. who introduced the White Esthetic Score (WES) that reflects the esthetic outcome with regard to the quality of the implant crown [8]. These evaluation methods have since been used by researchers who aim to more objectively evaluate and report esthetic outcomes of implant restorations particularly in the anterior maxilla [10].

Patient satisfaction, which indicates the success of the implant treatment from the patient’s perspective, is another important outcome measure and is commonly performed with questionnaires or a visual analog scale (VAS) [2, 8]. A current review of the literature, however, reveals only a limited number of studies reporting on patient-centered outcomes in addition to objective evaluations of implant-supported rehabilitations in the anterior maxilla. Among these, few studies previously reported that objective assessments may fail to reflect subjective patient outcomes [11–13]. Therefore, this study aims to investigate and report objective and patient-reported esthetic outcomes and how well they correlate for single-tooth implant restorations in the maxillary anterior region.

## Methods

This study was conducted in accordance with the Declaration of Helsinki on medical protocol and was approved by the Akdeniz University Ethical Review Board.

### Patient selection

**Fig. 1:**
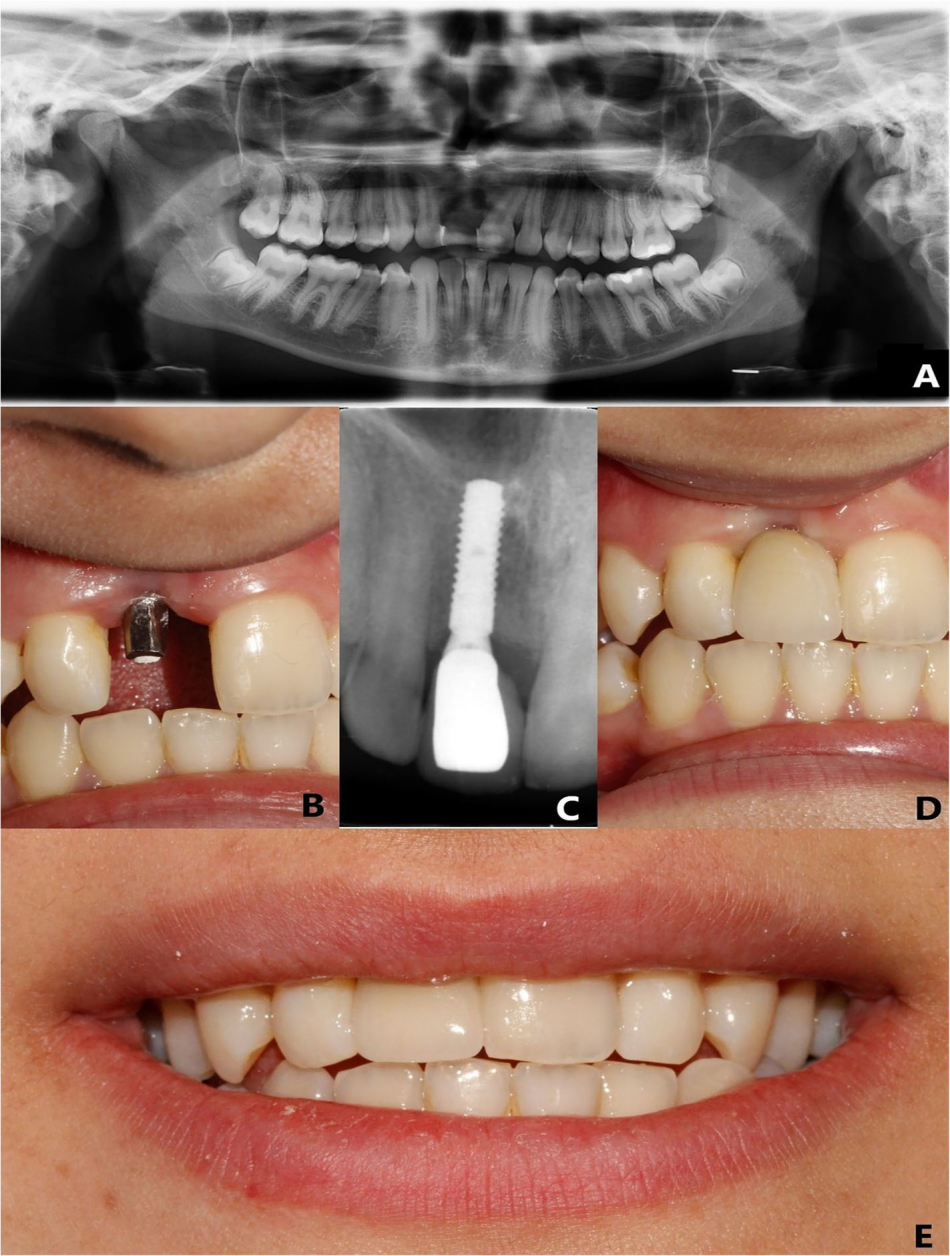
**a** Pre-operative panoramic radiograph of the patient, (**b**) abutment in place, following the osseointegration period, (**c**) periapical radiograph at 1-year follow up, (**d**) final restoration at 1-year follow up, and (**e**) smile line

The patients rehabilitated with a single implant-supported fixed prosthesis in the maxillary esthetic zone at the departments of Oral and Maxillofacial Surgery and Prosthetic Dentistry of Akdeniz University between June 2015 and April 2017 were included in this study. Maxillary esthetic zone was considered from and including canine to canine. All patients included in the study had only one missed tooth, rehabilitated with dental implant, in the esthetic zone, and all adjacent teeth were natural (Fig. 1). Patients with parafunctional habits, multi-unit restorations, restored contralateral tooth, or acute infection at the implant region were excluded. Patients that met the criteria were recalled for an esthetic outcome evaluation and given a subjective assessment questionnaire.

### Examination protocol

Demographic information of each patient along with the details of the intraoral evaluation to assess the gingival biotype and the radiographic evaluation, and details regarding implants and surgical procedures were obtained from hospital records.

**Fig. 2:**
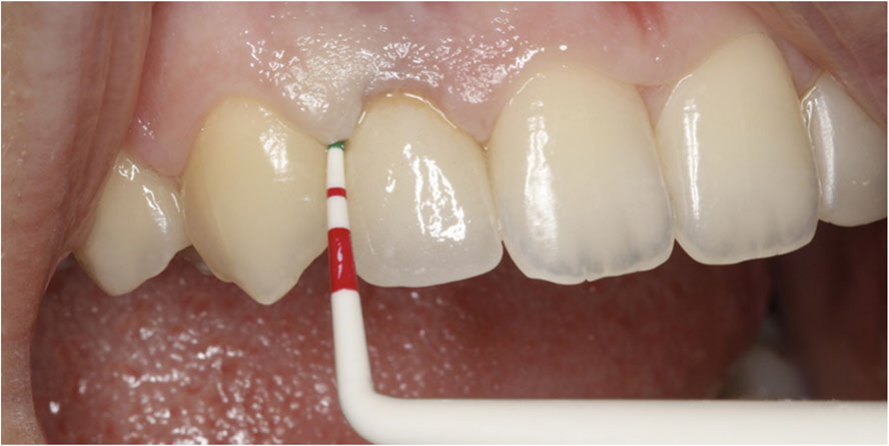
Probing of the peri-implant sulcus

Peri-implant tissues were examined with a Williams probe to determine the inflammatory status and the peri-implant sulcus depth. Gingival biotypes were recorded and classified as thick or thin using a periodontal probe (Fig. 2). The smile lines of each patient were observed during function (i.e., talking or smiling) and categorized as low, medium or high, according to the criteria set by Tjan et al. [14]. Digital periapical radiographs were obtained using the paralleling technique and Planmeca Romexis dental imaging software (Helsinki, Finland) was used to measure the distance between implant shoulder and alveolar crest.

### Esthetic assessment

#### Pink esthetic score

**Fig. 3:**
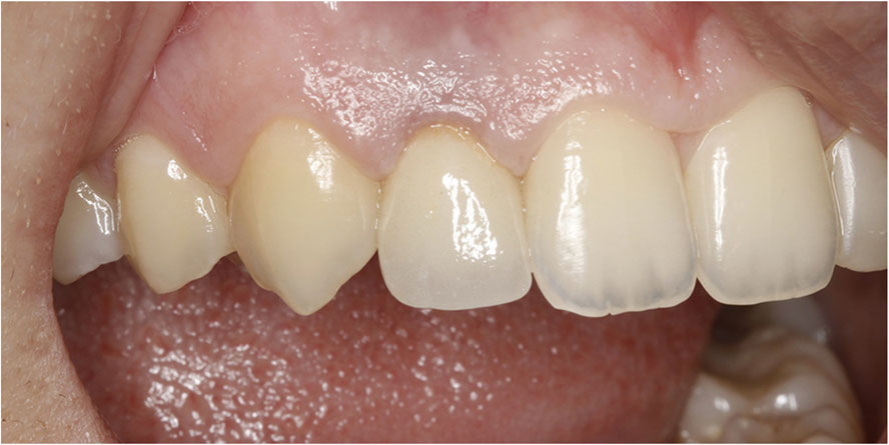
Vestibular view of right lateral implant supported crown

All patients were evaluated according to the pink esthetic score [9] which comprised the assessment of seven variables including the mesial papilla, distal papilla, soft tissue level, soft tissue contour, alveolar process deficiency, soft tissue color, and soft tissue texture (Fig. 3). Each variable was given a score of 0, 1, or 2. A score of 0 indicated the worst and a score of 2 indicated the best result for each variable, therefore the highest possible score of 14 denoted perfect peri-implant soft tissues. The threshold for clinically acceptable soft tissues was set at 8. A score of 12 or higher was accepted as almost perfect peri-implant soft tissues as previously described by Fürhauser et al. [9]

#### White esthetic score

**Fig. 4:**
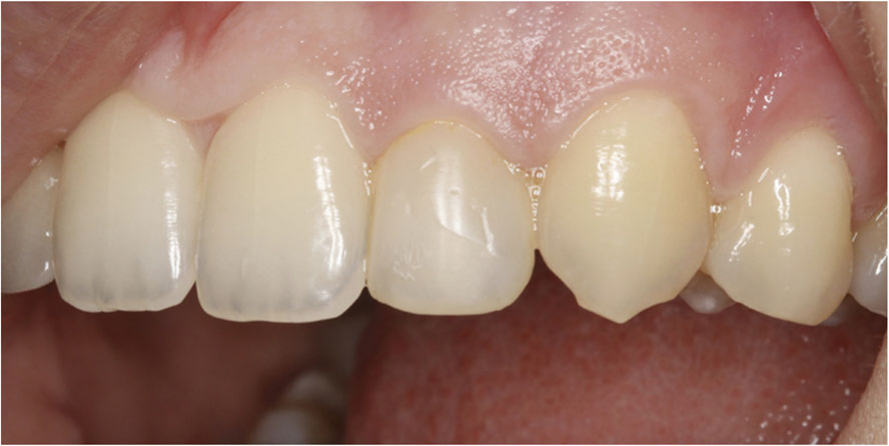
Vestibular view of contralateral lateral incisor

All patients were assessed according to the White Esthetic Score [8] which comprised the evaluation of five variables including general tooth form, tooth contour, tooth color (hue and value), surface texture, and translucence. Each variable was given a score of 0, 1, or 2. A score of 0 indicated the worst and a score of 2 indicated the best result for each variable. The implant-supported tooth was compared with the contralateral reference tooth in order to evaluate white esthetics (Fig. 4). A maximum score of 10 was given when the best mimicry of the contralateral tooth was achieved. The thresholds for a clinically acceptable or an almost perfect implant crown were set at 6 and 9 respectively.

### Patients’ perspective

Each patient was asked to rate their satisfaction regarding the overall implant treatment using a subjective outcome questionnaire. The questionnaire included five questions and a 10-cm visual analogue scale (VAS) labeled from 0 (worst possible result) to 10 (best possible result) to assess patient satisfaction. A mean VAS score was calculated for each patient based on their answers to the following questions.

1. How do you feel about the shape of your new implant tooth?
2. How do you feel about the color of your new implant tooth?
3. How do you feel about the shape of the gum that is around your new implant tooth?
4. How do you feel about the color of the gum that is around your new implant tooth?
5. What is your overall satisfaction with the new implant tooth?

### Statistical analysis

Data were collected and analyzed according to the inclusion and exclusion criteria. The statistical analysis was performed using IBM–SPSS version 22.0 (IBM Corp, NY, USA). The type of statistical analysis was determined according to the normality of the data. Bivariate analysis using Spearman’s correlation test was utilized between VAS and PES, VAS and WES, and VAS and smile line. Associations between gingival biotypes and PES, and gingival biotypes and WES were studied using Mann-Whitney *U* test. Associations between placement protocols and PES, and placement protocols and WES were studied using Kruskal-Wallis test. A multivariate evaluation was performed using regression analyses. *P* values of < .05 were used to assess the significance for all statistical analyses.

## Results

A total of 19 (7 female and 12 male) patients, who were rehabilitated with a single implant in the anterior maxilla, were included in this study. Patients’ ages ranged between 19 and 42 with a mean of 31.8 years. None of the implants were associated with increased probing depth, bleeding, suppuration, foreign body sensation, pain, morbidity, or infection. Clinical features related to anterior single implants are shown on Table 1.

**Table 1:** Clinical features of the implants

Implant site	Biotype	Smile line	Placement timing	PES	WES
12	Thick	Low	Type 3	12	9
13	Thick	Medium	Type 4	12	9
11	Thin	Medium	Type 4	6	9
23	Thick	Medium	Type 4	11	8
12	Thick	High	Type 2	8	9
12	Thin	High	Type 2	11	8
21	Thick	Medium	Type 3	12	10
21	Thin	High	Type 2	5	8
13	Thick	Medium	Type 3	11	8
13	Thin	Low	Type 4	12	9
23	Thick	Medium	Type 4	11	8
22	Thick	Low	Type 3	12	9
23	Thick	Medium	Type 2	12	8
13	Thick	Low	Type 4	9	9
12	Thick	High	Type 2	13	9
13	Thick	Medium	Type 4	12	8
11	Thin	Medium	Type 4	12	9
23	Thin	High	Type3	13	9
12	Thick	Medium	Type 4	11	8

*PES* pink esthetic score, *WES* white esthetic score

### Esthetic outcomes

The mean PES in this study was 10.7 (range 5–13, SD ± 2.24), which translated into an acceptable esthetic outcome. The mean WES was calculated as 8.6 (range 8–10, SD ± 0.60), which translated into an almost perfect outcome. All 19 implants received a WES of 6 or above, which was set to be the lowest threshold of clinical acceptance. Table 2 shows sub-classifications of PES and WES results and their corresponding percentages within this patient group.

**Table 2:** Esthetic scores, sub-classification of the results

	Number of patients	Percentage
Pink esthetic score		
Poor (0–7)	2	10.5%
Acceptable (8–11)	7	36.8%
Almost perfect (12–14)	10	52.6%
White esthetic score		
Poor (0–5)	0	0%
Acceptable (6–8)	8	42.1%
Almost perfect (9–10)	11	52.6%

Table 3 displays the results of PES and WES analyses in detail.

**Table 3:** Detailed results of pink and white esthetic score analyses

	Esthetic score		
	0	1	2
PES			
Mesial papilla	0	6	13
Distal papilla	3	6	10
Soft tissue level	0	6	13
Soft tissue contour	0	10	9
Alveolar contour	2	11	6
Soft tissue color	0	9	10
Soft tissue texture	0	2	17
WES			
Tooth form	0	9	10
Tooth contour	0	7	12
Color	0	1	18
Surface texture	0	6	13
Translucency	0	4	15

*PES* pink esthetic score, *WES* white esthetic score

### Visual analogue scale and subjective outcome questionnaire

The overall mean value of VAS was found to be 8.54 ± 0.36 (range 3.8–9.8). Mean and median values, as well as the range of scores for each question are given below:

- Question1. “How do you feel about the shape of your new implant tooth?” Mean patient rating was calculated as 8.5 (range 4–10, SD ± 1.8). Median score was 9. Thirteen patients responded with a score of ≥ 8 and 18 patients responded with a score of ≥ 6.
- Question2. “How do you feel about the color of your new implant tooth?” Mean patient rating was calculated as 8.5 (range 6–10, SD ± 1.3). Median score was 9. Thirteen patients responded with a score of ≥ 8 and 19 patients responded with a score of ≥ 6.
- Question3. “How do you feel about the shape of the gum that is around your new implant tooth?” Mean patient rating was calculated as 8.32 (range 2–10, SD ± 1.9). Median score was 9. Sixteen patients responded with a score of ≥ 8 and 17 patients responded with a score of ≥ 6.
- Question4. “How do you feel about the color of the gum that is around your new implant tooth?” Mean patient rating was calculated as 8.4 (range 2–10, SD ± 2.0). Median score was 9. Fifteen patients responded with a score of ≥ 8 and 17 patients responded with a score of ≥ 6.
- Question5. “What is your overall satisfaction with the new implant tooth?” Mean patient rating was calculated as 8.8 (range 5–10, SD ± 1.6). Median score was 9. Sixteen patients responded with a score of ≥ 8 and 17 patients responded with a score of ≥ 6.

Spearman’s correlation test failed to show any significant associations between the overall mean VAS results and PES or WES (*p* = 0.475, *p* = 0.984, respectively) (Table 4).

**Table 4:** Spearman’s correlation test between VAS and PES or WES

			PES	WES	VAS
Spearman’s rho	VAS	Correlation coefficient	− .174	− .005	1.000
		Sig. (two-tailed)	.475	.984	.
		*N*	19	19	19

*VAS* visual analogue scale, *PES* pink esthetic score, *WES* white esthetic score

### Gingival biotype

Majority of the patients (13 out of 19) were found to have thick gingival biotype and 6 patients had thin gingival biotype. When these patients were evaluated according to their gingival biotypes, those with thin and thick biotypes had mean PES values of 9.83 (range 5–13, SD ± 3.43) and 11.23 (range 8–13, SD ± 1.36), respectively. WES for thin and thick-biotyped patients were found to be 8.67 (range 8–9, SD ± 0.52) and 8.62 (range 8–10, SD ± 0.65), respectively. PES and WES scores for each group of patients (i.e., thin and thick biotypes) failed to show any statistically significant difference (*p* = 0.701, *p* = 0.831; Mann-Whitney *U* test).

### Smile line

Low, medium, or high smile lines were determined in four, ten, and five patients, respectively (Table 5).

**Table 5:** PES of patients with different smile lines

PES result	Smile line		
	Low (*n* = 4)	Medium (*n* = 10)	High (*n* = 5)
Poor (0–7)	0	1	1
Acceptable (8–11)	1	4	2
Almost perfect (12–14)	3	5	2

*PES* pink esthetic score

There was a significant negative correlation between the smile line and VAS; meaning that patients with lower smile lines expressed higher patient satisfaction (*p* = .001, Spearman’s correlation analysis) (Table 6).

**Table 6:** Spearman’s correlation test between smile line and VAS

		Smile line	VAS
Smile line	Correlation coefficient	1	− .699^a^
	Sig. (two-tailed)		.001
	*N*	19	19

*VAS* visual analogue scale

^a^Correlation is significant at the 0.01 level (two-tailed)

### Placement protocol

Type 2 (early, with soft tissue healing) and type 3 (early, with partial bone healing) placement protocols were followed for five implants each, whereas type 4 (late, with complete bone healing) placement was the most common protocol accounting for nine of the implants. Immediate implant placement (type 1) was not utilized in any patient. PES and WES for different placement protocol groups (i.e., type 2, 3, or 4) failed to show any statistically significant difference (*p* = 0.296, *p* = 301 respectively; Kruskal-Wallis test).

### Loading protocol

All implants were loaded 3–6 months after implant surgery (conventional loading protocol); therefore, this parameter was not analyzed in this study.

### Multivariate analyses

The overall effect of gingival biotype and smile line on PES, WES, or VAS was studied using a general linear regression analysis for multivariate tests. Although both PES and WES were not affected (*p* = 0.580, *p* = 303; respectively), VAS was significantly affected by both gingival biotype and smile line (*p* = 0.009). Accordingly, smile line had a stronger impact on VAS than the gingival biotype (*p* = 0.002, *p* = 0.492, respectively). The VAS of patients with high and low, and high and medium smile lines differed significantly (*p* = 0.004, *p* = 0.002; respectively), while medium and low smile lines did not show significant variation (*p* = 0.777). High smile line had a stronger influence on VAS than low and medium smile lines.

## Discussion

Ever since the introduction of dental implants in the 1960s, they have been used worldwide with high success rates and accepted predictability [13]. Initial efforts of implant treatment mainly focused on osseointegration and function, whereas today, esthetics is also regarded as an essential component, which is commonly addressed together with functional goals of rehabilitation with dental implants [15]. In the anterior maxilla, the definition of success embodies several factors in addition to absence of pain, bleeding, or other morbidities [3].

Pink and white esthetic scores (PES and WES) were developed in an attempt to allow objective evaluation of esthetics in implant dentistry [8, 9]. However, esthetic outcomes should measure both an objective assessment by the clinician and a subjective evaluation by the patient [13]. Only a limited number of studies have previously reported on the correlation between the objective evaluation of implants placed in the esthetic zone with patient-reported outcomes [2, 10, 13, 15–17].

The results of this study revealed acceptable outcomes in both PES and WES analyses. The mean PES in this study was 10.7, a score that is similar to the one previously reported by Cosyn et al. and higher than those from several other studies [2, 10, 16, 18]. The WES results on the other hand revealed an almost perfect outcome with a mean of 8.6, which was similar to studies by Angkaew et al. and Beekmans et al. and higher than the ones reported by Cho et al. and Gjelvold et al. [2, 10, 15, 16]. Even though variable overall PES and WES were reported on different studies, on a clinical basis, these indices not only reveal a patient-specific objective evaluation for the esthetic outcome but may aid in record-keeping and over-time assessment of anterior implant treatment. Angkaew et al. who also used VAS to determine patients’ perception of the outcomes reported higher VAS scores than our findings, although they found acceptable but lower PES and WES values. It is noteworthy that, as in the present study, they found no significant relationship between PES/WES and VAS scores [2]. On the contrary, similar results in VAS scores were achieved by Cho et al. who reported poor PES and almost acceptable WES results with a statistically significant correlation of VAS [10]. The outcomes of this study did not reveal a significant correlation between PES/WES results and patient satisfaction, which may be attributed to subjective nature of esthetics and the incompatibility of patient perspective with what is considered as an “objectively perfect clinical result”. Therefore, when our findings are considered together with the previous literature, it can be concluded that the outcomes from objective evaluations may not always fall in line with the patients’ satisfaction levels. An implant with perfect pink and white esthetic scores may in fact prove to be unsatisfactory for the patient, which emphasizes the significance of subjective evaluation by the patient when assessing esthetic outcomes.

In the present study, only two implants in patients with thin gingival biotypes failed to reach the clinically acceptable PES level. All other patients with both thick and thin biotypes achieved either acceptable or almost perfect scores for both PES and WES. According to the results of Angkaew et al., the PES/WES scores of patients with thick gingival biotype were significantly higher than those with thin gingival biotype; although no such correlation was detected in this study [2]. Peri-implant soft tissue stability is reported to be associated with gingival biotype, which is a significant parameter for the esthetic outcome of the implant restoration in the anterior region [19–21]. However, the results from the present study demonstrate that the gingival biotype may not necessarily play a very central role on esthetics in single implant restorations. Therefore, the authors of this study believe that patients with thin gingival biotypes may also expect to have acceptable esthetic results for single implants in the anterior maxilla so long as proper surgical and prosthetic protocols are meticulously designed and implemented.

The smile line is an indispensable element of esthetic dentistry and an important consideration of any oral rehabilitation. A recent study by Antoniazzi et al. among Brazilian population reported that patients with high smile lines were significantly less satisfied with their smile compared to those with lower smile lines [22]. Even though their study consisted of patients with natural teeth, the analyses from this study similarly found a significant negative correlation between VAS and the smile line of patients with anterior implant restorations. Accordingly, the patients with low and medium smile lines had similar levels of concerns regarding the esthetic aspects of their implants. However, the patients with high smiles lines and significantly lower VAS scores had worse perceptions than both groups. Therefore, patients with lower smile lines more frequently reported favorable esthetic outcomes. Patients with high smile lines are considered a challenge in attaining esthetic results, since the restoration and the gingival tissues are displayed more than those with lower smile lines, where these elements are hidden behind the upper lip [23, 24]. Thus, smile line evaluation should not be skipped during implant consultations in order to accurately meet patient expectations.

Only a limited number of studies compared esthetic outcomes of implants placed using different protocols [3, 25, 26]. A majority of these studies compared the results of immediate implant placement (type 1) with other protocols. Huynh-Ba et al. found no difference in terms of esthetics between type 1 and type 2 implant placement [25]. Similarly, Boardman et al. observed higher PES results following immediate placement, although not reaching statistical significance [3]. Another study, which reported on patient-related outcomes of immediately loaded single implants in the anterior maxilla, also revealed no statistically significant differences between PES and WES of implants that followed immediate (type 1) and late (type 4) placement protocols [26]. Although no patients in this study underwent immediate implant placement, favorable outcomes in terms of PES, WES, and VAS scores were achieved with type 2, 3, or 4 placement protocols among which no statistically significant difference was found. As for the loading protocols, all implants in this study underwent conventional loading due to a high level of patient compliance, lesser tendency for implant loss, and improved implant survival compared to immediate loading [27]. However, it should be noted that the literature suggests no statistically significant differences between PES and WES outcomes for immediate and delayed loading protocols [28].

The limitations to generalization of the findings of this study include limited sample and errors inherent to retrospective nature of the study. Further studies with larger sample sizes may aid in identifying the efficacy of objective assessment methods in evaluating esthetic outcomes in the anterior maxilla and how they relate to patient-related outcomes. Also, longer follow-ups are required to thoroughly assess the accuracy of PES/WES changes over time to predict any future peri-implant differences. Despite these limitations, the findings of this study provide an additional insight to how the perception of esthetics is affected in the presence of single implants in the anterior maxilla. Moreover, if validated by further research, PES/WES indices may gain ground in routine clinical practice to monitor long-term alterations of single implant restorations and peri-implant soft tissues.

## Conclusions

Professionally reported esthetic outcomes (PES and WES results) may not significantly correlate with patient-reported outcomes, although they are helpful in monitorization of implants in the anterior zone during follow-ups. This study reveals that smile line is a significant factor in patient satisfaction, which should be evaluated thoroughly prior to implant placement in the anterior maxilla.

## Abbreviations

PES: Pink esthetic score
SD: Standard deviation
SPSS: Statistical Package for the Social Sciences
VAS: Visual analogue scale
WES: White esthetic score
